# Chlorpyrifos Oxon-Induced Isopeptide Bond Formation in Human Butyrylcholinesterase

**DOI:** 10.3390/molecules25030533

**Published:** 2020-01-25

**Authors:** Kevser Biberoglu, Ozden Tacal, Lawrence M. Schopfer, Oksana Lockridge

**Affiliations:** 1 Department of Biochemistry, School of Pharmacy, Hacettepe University, Ankara 06230, Turkey; kevserb@hacettepe.edu.tr (K.B.); tacal@hacettepe.edu.tr (O.T.); 2 Eppley Institute, University of Nebraska Medical Center, Omaha, NE 68198-5900, USA; olockrid@unmc.edu

**Keywords:** mass spectrometry, isopeptide, chlorpyrifos oxon, crosslinked peptides, butyrylcholinesterase

## Abstract

A newly recognized action of organophosphates (OP) is the ability to crosslink proteins through an isopeptide bond. The first step in the mechanism is covalent addition of the OP to the side chain of lysine. This activates OP-lysine for reaction with a nearby glutamic or aspartic acid to make a gamma glutamyl epsilon lysine bond. Crosslinked proteins are high molecular weight aggregates. Our goal was to identify the residues in the human butyrylcholinesterase (HuBChE) tetramer that were crosslinked following treatment with 1.5 mM chlorpyrifos oxon. High molecular weight bands were visualized on an SDS gel. Proteins in the gel bands were digested with trypsin, separated by liquid chromatography and analyzed in an Orbitrap mass spectrometer. MSMS files were searched for crosslinked peptides using the Batch-Tag program in Protein Prospector. MSMS spectra were manually evaluated for the presence of ions that supported the crosslinks. The crosslink between Lys544 in VLEMTGNIDEAEWEWK_544_AGFHR and Glu542 in VLEMTGNIDEAEWE_542_WK satisfied our criteria including that of spatial proximity. Distances between Lys544 and Glu542 were 7.4 and 9.5 Å, calculated from the cryo-EM (electron microscopy) structure of the HuBChE tetramer. Paraoxon ethyl, diazoxon, and dichlorvos had less pronounced effects as visualized on SDS gels. Our proof-of-principle study provides evidence that OP have the ability to crosslink proteins. If OP-induced protein crosslinking occurs in the brain, OP exposure could be responsible for some cases of neurodegenerative disease.

## 1. Introduction

We dedicate our manuscript to Dr. Elsa Reiner, an outstanding scientist with whom we enjoyed collaborations on cholinesterase kinetics. Years ago, Dr. Patrick Masson, an expert in enzyme kinetics, gave us (O.L. and L.M.S.) a precious gift, the book by W.N. Aldridge and Elsa Reiner entitled “Enzyme Inhibitors as Substrates. Interactions of esterases with esters of organophosphorus and carbamic acids”. This book has served as an important reference work. Based on the information in this book, the scientific community is developing therapeutic applications for cholinesterases and cholinesterase inhibitors. Cholinesterases protect from the toxicity of organophosphorus nerve agents and pesticides by destroying these poisons before they interfere with nerve impulse transmission. Inhibitors are being tested for treatment of Alzheimer’s disease. The work of Elsa Reiner is indispensable in these ongoing efforts.

In 2014 Schmidt et al. reported that V-type, organophosphonate, chemical-warfare agents spontaneously formed covalent complexes with lysine residues on ubiquitin and that these complexes could react further to spontaneously form isopeptide linkages with neighboring glutamates [1]. Subsequently, it was shown that aggregation of proteins via isopeptide crosslinking could be induced by the pesticide-related organophosphate chlorpyrifos oxon ethyl (CPO) [2,3]. Proteins affected included casein, serum albumin, butyrylcholinesterase, and tubulin.

Accumulation of proteins in aggregates is a characteristic feature of neurodegenerative diseases such as Parkinson’s and Alzheimer’s. Stabilization of intraneuronal protein aggregates by transglutaminase-mediated, isopeptide crosslinking between ubiquitin and HSP27 protein was reported by Nemes et al. These authors correlated protein aggregate formation with Alzheimer’s disease and suggested that isopeptide crosslinking may play a role in the development of Alzheimer’s disease [4]. Immunofluorescence studies with antibodies directed at the isopeptide bond identified more immunoreactive neurons in Alzheimer’s disease cortex compared to controls [5,6].

The same gamma-glutamyl epsilon lysine isopeptide bond that is produced by the action of transglutaminase is produced by exposure to chlorpyrifos oxon [2,3]. Though the product isopeptide bond is identical, the residues that make the isopeptide bond are different. Transglutaminase makes an isopeptide bond between glutamine and lysine, whereas CPO makes an isopeptide bond between glutamate and lysine, or aspartate and lysine. Both the enzymatic and chemically-induced crosslinks cause proteins to aggregate. Exposure to organophosphorus pesticides is correlated epidemiologically to Alzheimer’s and Parkinson’s disease [7,8]. CPO-induced isopeptide crosslinking of proteins might be a link between pesticide exposure and the development of some cases of Alzheimer’s and Parkinson’s diseases.

To determine whether organophosphates other than chlorpyrifos oxon ethyl could promote isopeptide crosslinking and protein aggregation, the pesticide-related organophosphorus compounds chlorpyrifos ethyl, methamidophos, paraoxon ethyl, diazinon, diazoxon, monocrotophos, and dichlorvos were tested on human butyrylcholinesterase (HuBChE). Protein aggregation was visualized on SDS gels. We found that CPO had the most pronounced protein aggregation effect, while paraoxon ethyl, diazoxon, and dichlorvos had weak effects. Bands of BChE dimers (170 kDa), trimers (250 kDa), and aggregates at about 500 kDa resulted from treatment with 1.5 mM CPO. Aggregates up to tetramers (340 kDa) could be explained by isopeptide bond formation between Lys544 on one HuBChE monomer and Glu542 on another monomer. Formation of molecular weight aggregates larger than the tetramer can be explained by isopeptide crosslinks between tetramers.

## 2. Results and Discussion

### 2.1. CPO-Induced HuBChE Aggregation

**Figure 1:**
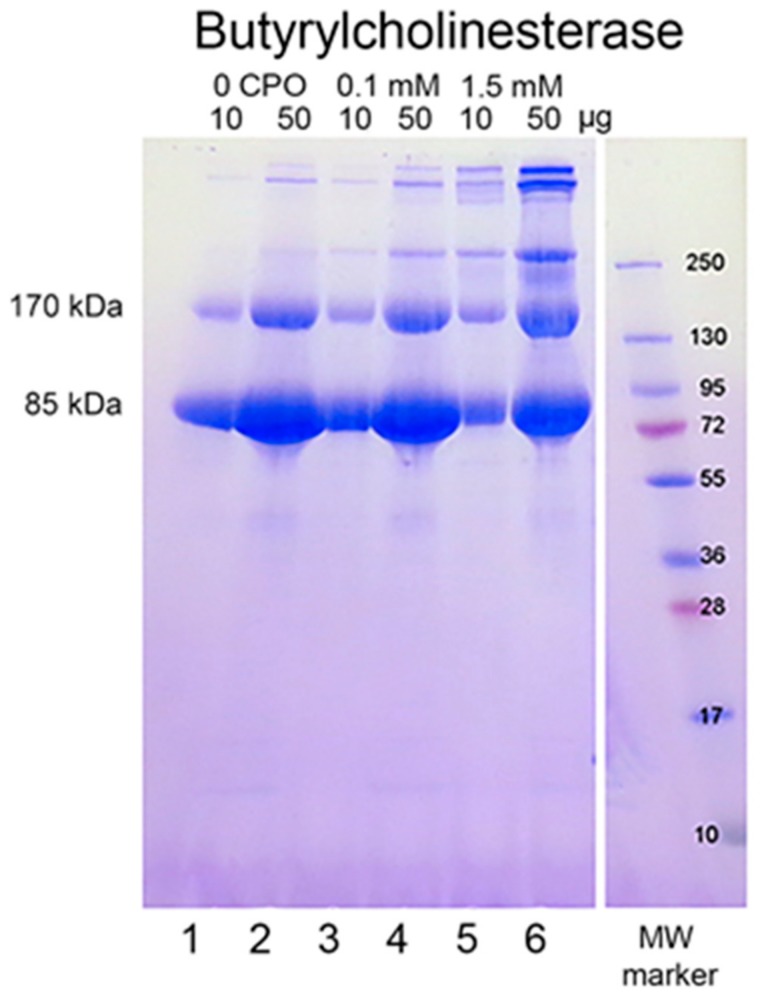
Chlorpyrifos oxon ethyl treated human butyrylcholinesterase. Lanes 1 and 2 contain 10 and 50 µg of untreated HuBChE, respectively. Lanes 3 & 4 contain 10 and 50 µg of HuBChE that was treated with CPO: 0.1 mM. Lanes 5 & 6 contain 10 and 50 µg of HuBChE that was treated with CPO: 1.5 mM. Lane 7 refers to the molecular weight markers.

When reduced HuBChE is run on an SDS polyacrylamide gel, it always shows a monomer band at 85 kDa and a non-reducible dimer band at about 170 kDa (Figure 1, lanes 1 & 2). Over time, during storage in phosphate buffered saline, at 4 °C, after filter sterilization, HuBChE develops additional non-reducible bands at about 250 kDa (trimer) and higher (doublet around 500 kDa) (Figure 1, lane 2). When the same HuBChE is treated with 1.5 mM CPO, the intensity of the bands at 170, 250, and around 500 kDa increases, at the expense of the 85 kDa monomer. In addition, new bands develop at about 230 kDa and in the 500 kDa range (Figure 1, lanes 5 & 6). A similar increase in band intensities develops following treatment with 0.1 mM CPO concentration (Figure 1, lanes 3 & 4).

### 2.2. Aggregate Formation Promoted by Other OP

**Figure 2:**
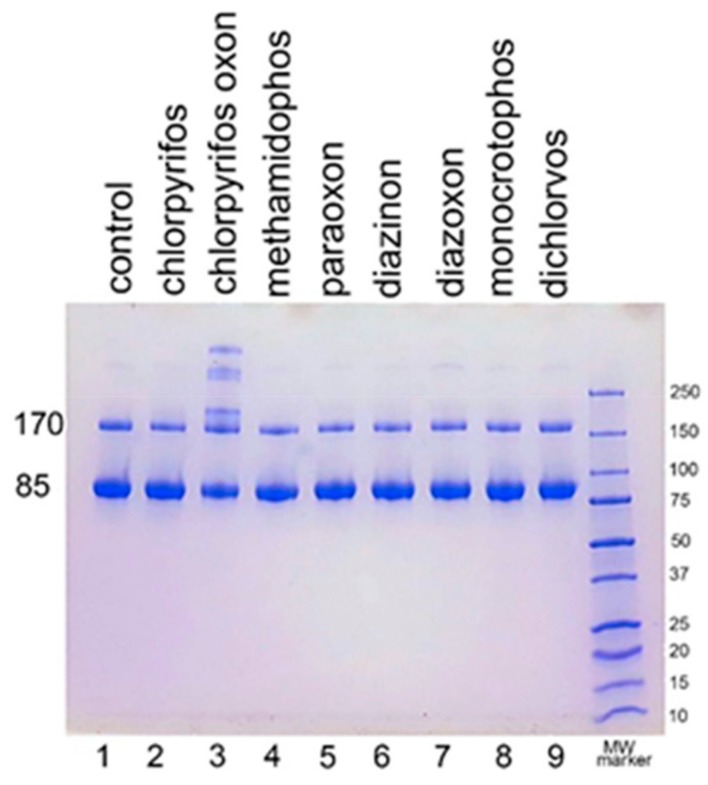
Treatment of human butyrylcholinesterase (HuBChE) with eight organophosphates (OP). Five µg of HuBChE were loaded per lane. Lanes 2–9 show the results for incubation of HuBChE with the indicated OP. Lane 1 shows the result for incubation without OP. Lane 10 refers to the molecular weight markers.

We examined the ability of other OP to induce aggregation. HuBChE was incubated at pH 8.5 and 37 °C for 7 days with 1.5 mM OP (chlorpyrifos ethyl, chlorpyrifos oxon ethyl, methamidophos, paraoxon ethyl, diazinon, diazoxon, monocrotophos, and dichlorvos). When the SDS gel was loaded with 5 µg HuBChE per lane, aggregation was evident only for CPO-treated HuBChE (Figure 2, lane 3). However, Figure S1 shows that SDS gels loaded with 50 µg HuBChE per lane exhibited an increase in band intensities for HuBChE treated with chlorpyrifos ethyl, chlorpyrifos oxon ethyl, paraoxon ethyl, diazoxon, and dichlorvos. The increase in band intensities was low compared to that induced by treatment with CPO.

### 2.3. Mass Spectral Identification of Isopeptide Crosslinks in CPO-Treated HuBChE

SDS gel bands at 85, 170, 250, and about 500 kDa for 1.5 mM CPO-treated HuBChE were cut from the gel (see Figure 1), digested with trypsin, and submitted for mass spectral analysis. The data were searched against the HuBChE sequence for the presence of isopeptide crosslinks between lysine and glutamate or lysine and aspartate, using the Batch-Tag (Web) and Search Compare algorithms from Protein Prospector v 5.22.1.

Crosslinked peptide candidates selected by Batch-Tag were checked to eliminate peptide pairs that were continuous in the primary HuBChE sequence. This type of artifact is unique to isopeptide crosslinks when the crosslinking element is loss of water. Loss of water can occur either during formation of isopeptide linkages or during formation of peptide linkages. Candidates involving the protein N-terminus were eliminated because the N-terminus of the HuBChE database entry starts at the leader sequence, which is not present in the mature protein. Sequences for the remaining candidates were manually evaluated.

Choosing a convincing crosslinked peptide relies on several subjective factors. The Batch-Tag Score reflects the statistical probability of a crosslink. For a crosslinked peptide a score of more than 20 is statistically significant. The Batch-Tag score difference is generally a measure of how much information there is on the less confident of the two peptides. With a simple sample such as a single pure protein, a small score difference may be satisfactory because the total parent ion mass is very restrictive (personal communication from Dr. R. Chalkley, Mass Spectrometry Facility at the University of California San Francisco). It is generally good to have sequence information from both peptides in the crosslink and it is helpful if most of the major peaks in the MSMS spectrum fit the proposed sequence. It is particularly convincing if a fragment that includes the crosslinked amino acids is observed. It is also necessary that the crosslinked residues exhibit spatial proximity (i.e., they are within a reasonable reaction distance as determined from the X-ray structure). For the purposes of this paper we are assuming that a reasonable distance is less than 10 Å.

**Figure 3:**
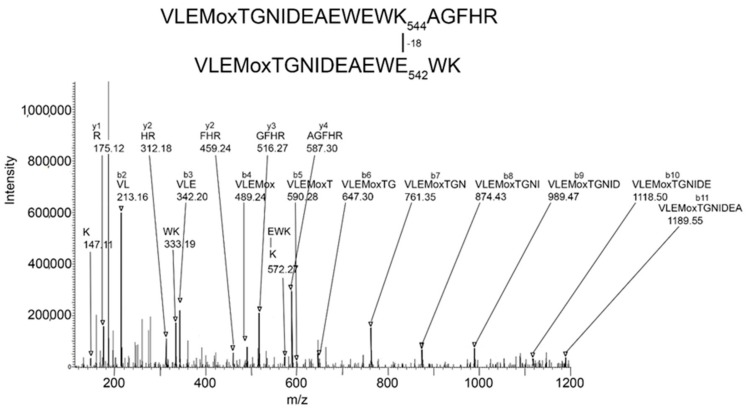
MSMS spectrum of peptide VLEMoxTGNIDEAEWEWK544AGFHR crosslinked to VLEMoxTGNIDEAEWE542WK through the crosslinking action of CPO. Annotated masses are from a b-ion sequence, and from two y-ion sequences that identify the crosslinked peptides. These peaks are darkened and denoted by an arrow. Most major peaks are part of these sequences. Most unannotated peaks are due to neutral losses of amine, water, or CO (a-ions). The parent ion mass is 897.01 *m*/*z*, charge state +5.

**Figure 4:**
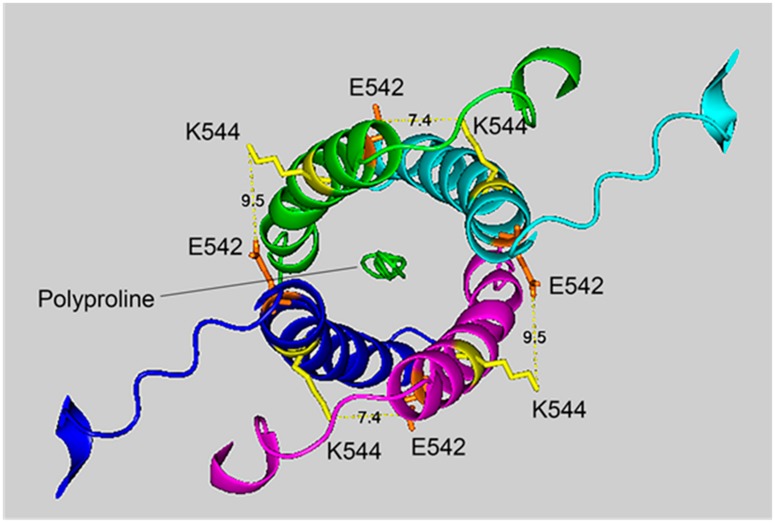
The crosslinking region of tetrameric HuBChE illustrating the location of the crosslinked residues for the peptide pair VLEMoxTGNIDEAEWEWK_544_AGFHR–VLEMoxTGNIDEAEWE_542_WK. The image shows the C-terminal α-helices from each monomer coiled into a superhelix, with a tetramer-forming polyproline II helix in the center. Lysines responsible for the crosslink are shown in yellow, while glutamates are orange. Potential bonds are indicated by dashed lines with the distance between residues given in Å. Deep-blue, purple, light blue and green identify α-helices from different HuBChE monomers.

**Figure 5:**
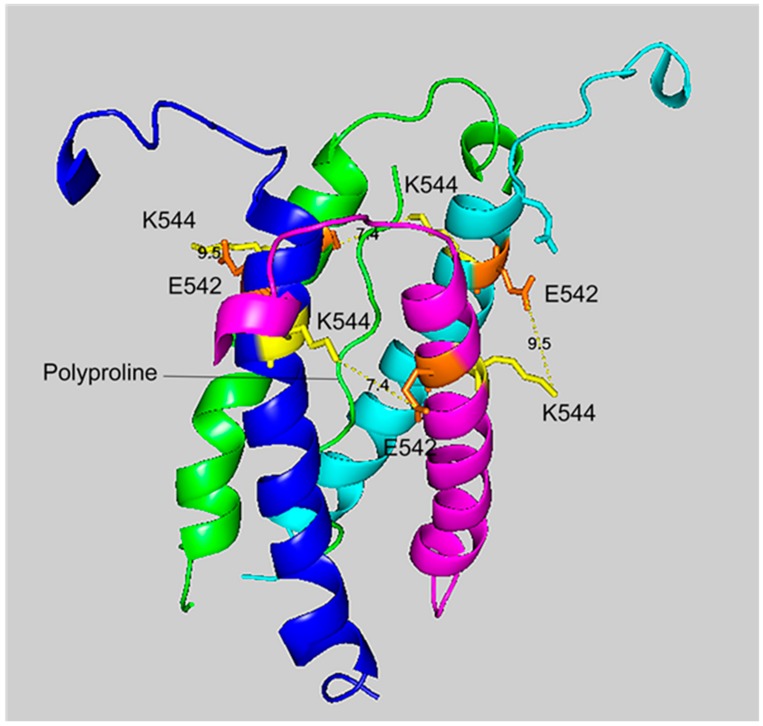
A side view of the crosslinking region of tetrameric HuBChE illustrating the location of the crosslinked residues for the peptide pair VLEMoxTGNIDEAEWEWK_544_AGFHR–VLEMoxTGNIDEAEWE_542_WK. The C-terminal alpha helices from the four HuBChE monomers are shown surrounding the tetramer forming polyproline II helix. Crosslinking glutamates are shown in orange. Crosslinked lysines are shown in yellow. The proposed crosslink distances of 9.5 Å and 7.4 Å are indicated with a yellow dashed line and the distance is given in Å. Deep-blue, purple, light blue and green identify α-helices from different HuBChE monomers.

Batch-Tag identified eight crosslinked candidates from the 500 kDa band, five from the 250 kDa band, three from the 170 kDa band, and five from the 85 kDa band. Of these, the crosslink between K544 and E542 in peptides VLEMoxTGNIDEAEWEWK_544_AGFHR and VLEMoxTGNIDEAEWE_542_WK was the only candidate that had all the factors which would make it convincing: score = 28–29, score difference = 4–6, sequence from both peptides, most of the major peaks in the MSMS spectrum fit to the structure, a fragment consistent with the crosslink was observed, and the distance between residues on different monomers was 7.4–9.5 Å. The other candidates had only 2–3 of the desirable factors. Consequently, they will not be considered in the following presentation. The VLEMoxTGNIDEAEWEWK_544_AGFHR peptide crosslinked to VLEMoxTGNIDEAEWE_542_WK appeared in data from all four SDS bands. The parent ion mass of 897.01 (charge state +5) is consistent with two peptides and rules out the possibility that the crosslink is within the linear sequence. The MSMS spectrum is shown in Figure 3 and a cryo-EM structure showing the location of the crosslinking residues is shown in Figure 4 and Figure 5.

The presence of the VLEMoxTGNIDEAEWEWK_544_AGFHR peptide crosslinked to VLEMoxTGNIDEAEWE_542_WK in the 85 kDa monomer band was unexpected. This crosslink requires that each peptide derives from a different protein monomer (i.e., they are part of a dimer). A dimer of 170 kDa is not expected in the 85 kDa monomer band of the SDS polyacrylamide gel. The solution to this dilemma is likely to be the well-known feature of SDS polyacrylamide gel separation wherein small amounts of proteins migrate at inappropriate positions. In the case of VLEMoxTGNIDEAEWEWK_544_AGFHR peptide crosslinked to VLEMoxTGNIDEAEWE_542_WK, quantitation by spectral counting revealed that there were 61 spectral counts in the 500 kDa band, 142 spectral counts in the 250 kDa band, 115 spectral counts in the 170 kDa band, but only 6 spectral counts in the 85 kDa band. Thus only 2% of the total amount of crosslinked protein appeared in the 85 kDa band. This observation supports the proposal that appearance of the VLEMoxTGNIDEAEWEWK_544_AGFHR peptide crosslinked to VLEMoxTGNIDEAEWE_542_WK in the 85 kDa band is an SDS polyacrylamide migration artifact.

### 2.4. Absence of the K544–E542 Crosslink in Control HuBChE

Control untreated HuBChE and CPO-treated HuBChE had bands at the same positions on an SDS gel as shown in Figure 1, though band intensities were weak in the control samples. Figure S1 shows that gel band intensities in control HuBChE were similar for samples stored at 4 °C and 37 °C. Gel slices for the control bands were cut out and analyzed for crosslinks using the protocol described for CPO-HuBChE gel slices. Control samples did not exhibit VLEMoxTGNIDEAEWEWK_544_AGFHR crosslinked to VLEMoxTGNIDEAEWE_542_WK. However, they did have other crosslinks. This study will be reported elsewhere.

### 2.5. MSMS Spectrum of VLEMoxTGNIDEAEWEWK_544_AGFHR Crosslinked to VLEMoxTGNIDEAEWE_542_WK

The MSMS spectrum of the VLEMoxTGNIDEAEWEWK_544_AGFHR–VLEMoxTGNIDEAEWE_542_WK crosslinked candidate (Figure 3) contains a b-ion sequence, VLEMoxTGNIDEA, which is consistent with the N-terminus from both peptides. There also is a y-ion sequence, AGFHR, from the C-terminal of the larger peptide, and a y-ion sequence, EWK, from the C-terminal of the smaller peptide. The y-ion sequence from the smaller peptide is linked to a mass at 572 *m*/*z* that is consistent with the crosslink fragment K (–18)EWK. The intensity of all signals in these sequences is at least two-times above background. There are two lysines in this crosslinked peptide that could potentially be involved in a crosslink. However, the lysine in the smaller peptide is part of a y-ion sequence, thereby disqualifying it from being the crosslink lysine. This leaves K544 in the larger peptide as the best candidate for the crosslink lysine. This assignment is supported by the crosslink fragment, K_544_(–18)EWK. There are five acidic groups in the smaller peptide that could serve as the acid partner in the crosslink. Three of these are part of the b-ion sequence, VLEMoxTGNIDE, thereby disqualifying them, to a first approximation, from being part of the crosslink. This leaves E540 and E542 as potential acid crosslinking partners. The crosslink fragment, K_544_(–18)E_542_WK constrains the crosslink partner to E542. However, other crosslinks between these peptides could occur.

If the VLEMoxTGNIDE, b-ion sequence is defined solely by residues in the larger peptide, then other glutamate residues in the smaller peptide could be available for crosslinking. In fact, Batch-Tag assigned a crosslink between K544 and E531. Manual evaluation of the MSMS spectrum for the potential K544-E531 crosslink in peptides VLEMoxTGNIDEAEWEWK_544_AGFHR–VLE_531_MoxTGNIDEAEWEWK could neither confirm nor refute this assignment. The distance between K544 and E531 is 6.3 Å, making a crosslink feasible. It is noteworthy that this crosslink can form only between two of the four possible pairs of monomers. This is due to an asymmetry in the tetramer structure. The distance between K544 and E531 in the other two pairs of monomers is 34.5 Å. Therefore, if a K544 to E531 crosslink does occur the product would only be dimers. Higher order aggregation is not possible.

### 2.6. Cryo-EM Structure of VLEMoxTGNIDEAEWEWK_544_AGFHR Crosslinked to VLEMoxTGNIDEAEWE_542_WK

Figure 4 and Figure 5 show different views of crosslink K544 to E542 in the HuBChE cryo-EM structure. The images are taken from cryo-EM data on the native HuBChE tetramer reported by Leung et.al [9], deposited in the Protein DataBase (PDB 6i2t). The images were created to emphasize the crosslinking region using the PyMOL Molecular Graphics System ver. 2.3.2 (Schrodinger).

The crosslinking residues are located in the C-terminal tetramerization domain of HuBChE. This region is composed of an α-helix from each monomer coiled into a superhelix which associates the monomers into a tetramer. The superhelix is locked in place by a polyproline II helix that fits into the center of the superhelix. The α-helix from each monomer is colored differently in the figures. The potential bonds between crosslinked residues are indicated by dashed lines and marked with the distance in Å. Two distances, 7.4 and 9.5 Å, are given because the HuBChE tetramer assembles as a quasi C2-symetric dimer of dimers in which the monomers are diagonally equivalent [9]. These HuBChE structures show that crosslinks between K544 and E542 are capable of forming dimers, trimers, and tetramers.

### 2.7. Mechanism of Isopeptide Bond Formation

**Figure 6:**
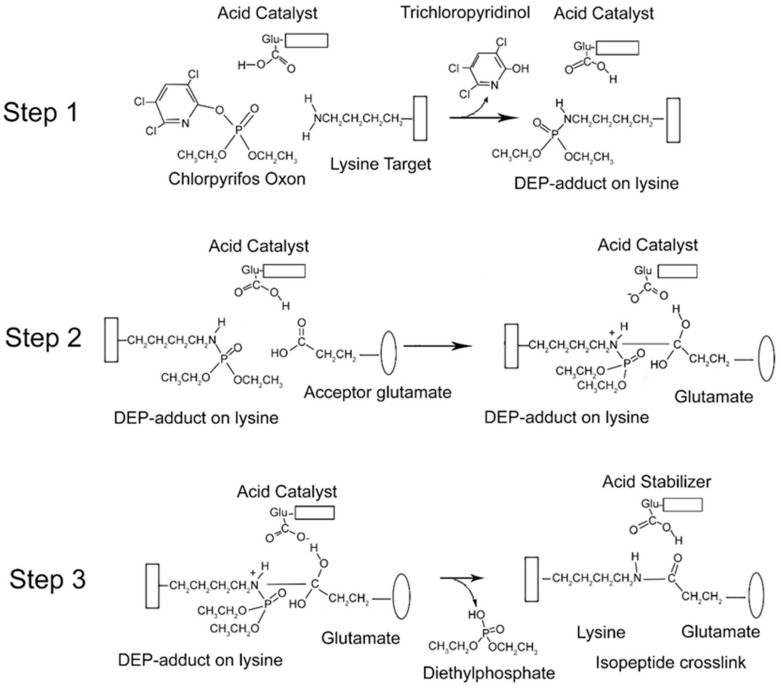
Mechanism for CPO-promoted isopeptide bond formation between lysine and glutamate. Step 1: diethylphosphate (DEP)-adduct formation on lysine catalyzed by a nearby glutamate. The box associated with the lysine represents protein. Step 2: isopeptide bond formation between the DEP–lysine and glutamate. The oval represents a different protein component. Step 3: release of the DEP moiety and stabilization of the isopeptide crosslink.

From the data presented above, it is clear that CPO promotes isopeptide bond formation. It has been argued that the mechanism for spontaneous formation of isopeptide bonds between lysine and glutamate or aspartate is catalyzed by nearby glutamate or aspartate residues [10,11]. A parallel mechanism has been proposed for CPO-promoted isopeptide bond formation [3]. The CPO-promoted reaction requires an initial reaction of CPO with the lysine destined to be included in the isopeptide bond. That reaction yields a diethylphospho-adduct. Formation of the DEP–lysine adduct is selective in that only a limited set of lysines undergoes the reaction. This suggests that adduct formation is catalyzed. An acidic catalyst is likely to be involved. In principle, the same acidic catalyst could promote both adduct formation and isopeptide bond formation. A mechanism for these processes is proposed in Figure 6.

There is an initial reaction of CPO with lysine to form a DEP-adduct (step 1 in Figure 6). The acid catalyst serves to accept a proton from the amine of lysine thereby making the nitrogen a better nucleophile, while at the same time donating a proton to the oxygen of the O–P bond of CPO, thereby increasing the positive character of the phosphorus and providing a proton for the hydroxyl of the trichloropyridinol upon its release. This is followed by a nucleophilic attack of the amine from the DEP–lysine adduct on the carbonyl carbon of the acceptor glutamate to form an isopeptide bond (step 2 in Figure 6). The acid catalyst is poised to deliver a proton to the carbonyl oxygen of the glutamate thereby making the carbonyl carbon more electrophylic, while at the same time it stabilizes the developing positive character of the lysyl amine. The final step is release of the DEP moiety (step 3 in Figure 6). The acid catalyst is in position to accept a proton from one hydroxyl of the glutamate thereby promoting the formation of the glutamate carbonyl and promoting the shift of the other glutamate hydroxyl to the phosphorus of the DEP. Neutralization of the positive charge on the lysyl nitrogen results in release of DEP. The final isopeptide bond is stabilized by hydrogen bonding with the former acid catalyst. This mechanism is comparable to that given by Kang and Baker for spontaneous isopeptide bond formation between lysine and aspartate [10]. The fact that spontaneous isopeptide bond formation between VLEMoxTGNIDEAEWEWK_544_AGFHR and VLEMoxTGNIDEAEWE_542_WK does not occur in BChE argues that there is something special about the intervention of the DEP adduct. It seems likely that DEP is a better leaving group than the hydroxyl in spontaneous isopeptide bond formation.

**Figure 7:**
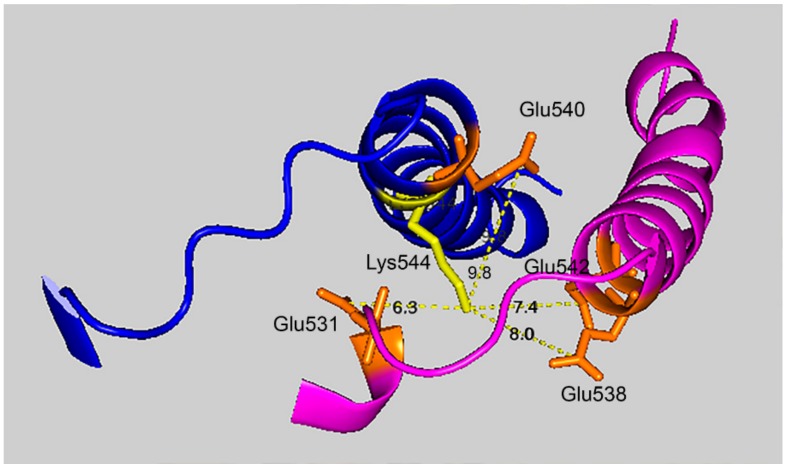
Glutamate promoted formation of the K544–E542 isopeptide bond. C-terminal α-helices from two HuBChE monomers are depicted along with the distances between Lys544 (yellow) and Glu542, Glu540, Glu538, and Glu531 (orange). Deep-blue, purple identify α-helices from different HuBChE monomers.

Figure 7 shows that the 7.4 Å crosslink between K544 and E542 is surrounded by glutamate residues that could promote DEP–lysine adduct formation and isopeptide bond formation. Glu531 is 6.3 Å from Lys544; Glu 538 is 8.0 Å away; and Glu540 is 9.8 Å away. Due to the asymmetry in the HuBChE structure there are two crosslink distances: 7.4 Å and 9.5 Å. The same catalytic glutamate residues are found near the 9.5 Å crosslink.

### 2.8. Diethylphosphate-Labeled Residues

Treatment of HuBChE with 1.5 mM CPO yielded 11 DEP-labeled lysines. Lysine 544 was one of these. This observation strengthens the case for the isopeptide bond between VLEMoxTGNIDEAEWEWK_544_AGFHR and VLEMoxTGNIDEAEWE_542_WK. In addition, there were 2 DEP-labeled serines, 10 DEP-labeled tyrosines, and 3 DEP-labeled threonines. A complete list of labeled peptides can be found in Table S1.

In addition to the two DEP-labeled serines, there were two MEP-labeled serines (monoethylphosphate also known as O-ethylphosphate) indicating the loss of an ethoxy group after initial formation of the DEP-labeled serine. One of these MEP-labeled serines was Ser198, the active-site serine of HuBChE. Based on spectral counts, 100% of the active-site serine was MEP-labeled. The high level of labeling was expected based on the high reactivity of Ser198 with organophosphorus compounds. That only MEP-adducts were seen is consistent with the known “aging” reaction of organophosphorylated HuBChE Ser198. This reaction removes one ethoxy group from the DEP-adduct, forming the MEP-adduct. The reaction is mediated by residues in the HuBChE active site.

Eight of the DEP–tyrosines were accompanied by MEP–tyrosine adducts. Two peptides contained only the MEP–tyrosine adduct. None of the DEP–Thr adducts were accompanied by MEP-adducts.

Overall, the fraction of labeled residues varied from 3% to 100%. Inspection of Table S1 shows many instances in which neighboring residues on the same peptide are identified as labeled. This likely reflects the inability of Protein Prospector (Batch-Tag) to discriminate between the options in those cases.

### 2.9. Aggregates Larger than Tetramers

Figure 1, lane 6, clearly shows that there are multiple bands in the 500 kDa range. This argues that HuBChE aggregates larger than the 340 kDa tetramer were created by treatment with CPO. Most of the crosslink candidates selected by Batch-Tag had distances between the lysine and glutamate/aspartate that were larger than 10 Å. One or more of these crosslinks could conceivably have arisen from linkages between tetramers to make the larger aggregates. Formation of large covalent aggregates would require non-covalent interaction between HuBChE tetramers prior to covalent crosslink formation.

### 2.10. Relative Reactivity of Organophosphates with Lysine

It was found that organophosphates other than chlorpyrifos oxon are less efficient in the crosslinking process. Since the crosslinking reactions for different organophosphates are identical, except for the initial adduct formation (step 1, see Figure 6), the difference in efficiency must be associated with that step. Step 1 is a nucleophilic attack of the ε-amine of lysine on the phosphorous of the organophosphate with release of the leaving group. Lower reactivity of the phosphothioates (chlorpyrifos ethyl and diazinon) can be attributed to the lower electrophilicity of the phosphorus, due to the double-bonded sulfur in contrast to double-bonded oxygen. Lower reactivity of the other compounds (methamidophos, paraoxon ethyl, diazoxon, monocrotophos, and dichlorvos) may be due to the acidity of the leaving group. March, on page 305 of his book [12], states that hydrolysis reactions are most successful when the ester is that of a strong acid. Hydrolysis reactions are analogous to the nucleophilic attack of the ε-amine of lysine on a diethyl organophosphate to form diethylphospho-lysine and release the leaving group. Based on relative pKa values for the leaving groups, chlorpyrifos oxon (pKa of trichloropyridinol = 4.6) is an ester of a stronger acid than either paraoxon ethyl (pKa of p-nitro phenol = 7.6) or diazoxon (pKa of 2-isopropyl-4-methyl-6-hydroxy pyrimidine = 9.7). Therefore, the rate of reaction of lysine with an OP correlates with the pKa of the OP leaving group. For example, it explains why chlorpyrifos oxon (pKa 4.6) reacts more rapidly than paraoxon ethyl (pKa 7.6).

## 3. Conclusions

Chlorpyrifos oxon promotes isopeptide bond formation in HuBChE, leading to dimer, trimer, and higher complexes. Other OP are less efficient in this process, however there is preliminary evidence for crosslinking promoted by paraoxon ethyl, diazoxon, and dichlorvos.

The most well-defined isopeptide crosslink between HuBChE monomers is K544 to E542 located in the C-terminal tetramerization region. Other, more poorly defined crosslinks are implicated in the formation of aggregates larger than the tetramer.

## 4. Methods and Materials

Human butyrylcholinesterase (HuBChE), accession P06276, was purified from frozen Cohn fraction IV-4, in-house [13] and stored as a sterile solution in phosphate buffered saline at 4 °C. The organophosphates (OP) used in this study were chlorpyrifos oxon ethyl (*O*,*O*-diethyl *O*-3,5,6-trichloropyridin-2-yl phosphate, CAS 5598-15-2 Chem Service, West Chester, PA, USA, cat# N11459B), chlorpyrifos ethyl (*O,O*-diethyl *O*-3,5,6-trichloropyridin-2-yl phosphorothioate, CAS 2921-88-2 Chem Service cat# N-11459), methamidophos (*O*,*S*-dimethyl phosphoramidothioate, CAS 102-92-6 Chem Service cat# N-12393), paraoxon ethyl (*O,O*-diethyl 4-nitrophenyl phosphate, CAS 311-45-5 Chem Service cat# N-12816), diazinon (O,O-diethyl 2-isopropyl-6-methyl-4-pyrimidinyl phosphorothioate, CAS 333-41-5 Chem Service cat# N11621), diazoxon (*O,O*-diethyl 2-isopropyl-6-methyl-4-pyrimidinyl phosphate, CAS 962-58-3 Chem Service cat# MET11621A), monocrotophos (*O,O*-dimethyl (*E*)-1-methyl-2-(methylcarbamoyl) vinyl phosphate, CAS 6923-22-4, Chem Service cat# N-12493), or dichlorvos (2,2-dichlorovinyl-dimethyl phosphate, CAS 62-73-7 Chem Service cat# N-11675). Stock solutions of 0.1 M OP were prepared in acetonitrile and stored at −80 °C.

### 4.1. Treatment of HuBChE with Organophosphorus Toxicants 

HuBChE 99% pure, 2.3 mg/mL was incubated with 0, 0.1, or 1.5 mM chlorpyrifos ethyl, chlorpyrifos oxon ethyl, methamidophos, paraoxon ethyl, diazinon, diazoxon, monocrotophos, or dichlorvos for 7 days in 10 mL of 50 mM Tris/Cl plus 0.1% sodium azide, pH 8.5 at 37 °C. To evaluate temperature effects, control HuBChE was incubated in pH 8.5 buffer at 4 °C and 37 °C. Excess OP was removed by dialysis against 4 × 4 L of 20 mM ammonium bicarbonate buffer pH 8.1 at 4 °C. Each sample was dialyzed in a cellulose dialysis bag (Sigma, St. Louis, MO, USA, cat# D9777) in a separate beaker with four changes of dialysis buffer, at 24 h intervals. Effectiveness of dialysis was assessed by testing the inhibitory potency of spent dialysis buffer at the end of each 24 h dialysis. The test consisted of incubating 200 µL of spent dialysis buffer with 200 µL of 1.1 unit/mL HuBChE for 30 min and assaying 100 µL of the incubation mixture for residual HuBChE activity. Residual activity from HuBChE after incubation with the spent dialysis buffer was compared to HuBChE activity after incubation with fresh buffer. The HuBChE activity assay was conducted in 2 mL 0.1 M potassium phosphate buffer pH 7.4 containing 0.5 mM dithio-bis-nitrobenzoic acid (Sigma, ≥98% BioReagent, cat# D8130) and 1 mM butyrylthiocholine (Fluka, ≥99%, cat# 20820) at 25 °C. Activity was detected by increase in absorbance at 412 nm recorded with a Gilford spectrophotometer.

After four buffer changes the spent dialysis buffers did not inhibit BChE, an indication that excess OP was dialyzed out of the BChE protein solutions. This precaution was considered essential because residual OP could complicate the interpretation of the reaction of OP with native HuBChE by reacting with denatured HuBChE or with tryptic peptides of HuBChE. OP are relatively stable, CPO being the most labile, having a half-life of 21 days in 20 mM Tris/Cl plus 0.1% sodium azide, pH 8 and 23 °C [14].

### 4.2. SDS Polyacrylamide Gel Electrophoresis

SDS 4-22% gradient polyacrylamide gels with a 4% stacking gel, 0.75 mm thick, were prepared in a Hoefer vertical slab gel unit (Hoefer Scientific Instruments, Model SE600, Holliston, MA, USA). OP-treated and control HuBChE samples were denatured in a boiling water bath for 3 min in the presence of 50 mM dithiothreitol (Fisher Biotech, Hampton, NH, USA, electrophoresis grade cat# BP172), 2% SDS (Sigma, ≥99%, electrophoresis reagent, cat# L3771), 0.05% bromophenol blue (Fisher BioTech, electrophoresis grade, cat# BP114), and 30% glycerol before electrophoresis. The sample wells in the Hoefer gels had a 90 µL capacity. Hoefer gels were loaded with 50 µg BChE per lane. Precast 4–15% gradient gels (BioRad, Hercules, CA, USA, cat#4568084) were loaded with 5 µg BChE per lane. Precision Plus Protein Standards (BioRad cat# 161-0373) were used for mass reference. Gels were stained with Coomassie blue R250 (Fisher Biotech, electrophoresis grade, cat# BP101) and destained with water.

### 4.3. In-Gel Tryptic Digestion of HuBChE Aggregates

Bands for CPO-treated HuBChE at 85 kDa, 170 kDa, 250 kDa, and about 500 kDa were cut from the SDS polyacrylamide gel and chopped into bits. Control bands at 85 kDa and 170 kDa from samples that were not treated with CPO were cut from separate gels and chopped into bits. Each band was processed separately. Coomassie blue was extracted from the gel bits with 200 µL aliquots of 50% acetonitrile (Fisher, synthesis grade, cat# BP1170) in 25 mM ammonium bicarbonate (Sigma, BioUltra, cat# 09830). The shrunken gel bits were dried in a vacuum centrifuge, resuspended in 200 µL of 0.1 M ammonium bicarbonate, reduced with 10 mM dithiothreitol, and alkylated with 55 mM iodoacetamide (Sigma cat# I6125). Excess reagent was washed out of the gel bits with 50% acetonitrile in 25 mM ammonium bicarbonate. The washed gel bits were dried by vacuum centrifugation. The dry gel bits were re-swollen with 150 µL of 25 mM ammonium bicarbonate containing 1.8 µg of sequencing grade trypsin (Promega, Madison, WI, USA, cat# V511C) and incubated overnight at 37 °C. Tryptic peptides in the supernatant were saved. The gel bits were incubated with 200 µL of 0.1% trifluoroacetic acid (Beckman, Indianapolis, IN, USA, sequencing grade cat# 290204) in 60% acetonitrile for 60 min to extract more peptides. The extract was combined with the first supernatant and the extraction was repeated. The pooled extracts were centrifuged for 30 min at 14,000× *g* to pellet any polyacrylamide particles before the supernatant was transferred to a new microfuge tube and dried by vacuum centrifugation. The dried peptides were dissolved in 20 µL of 0.1% formic acid (Fisher Scientific, Optima LC/MS cat# A117), and submitted for mass spectral analysis.

### 4.4. Mass Spectral Analysis of Tryptic Peptides

Mass spectral data were acquired on an Orbitrap Fusion Lumos mass spectrometer (Thermo Fisher Scientific, Waltham, MA, USA) connected to a Thermo RSL Ultimate 3000 ultra high pressure liquid chromatograph (Thermo Scientific). Two microliters of sample were loaded onto an Acclaim Pep Map 100 C18 trap column (75 µm × 2 cm; Thermo Scientific) and washed with 98% solvent A and 2% solvent B for 10 min at 4 µL/min. Solvent A was 0.1% formic acid in water. Solvent B was 80% acetonitrile and 0.1% formic. Flow was shunted onto the Thermo Easy-Spray Pep Map RSLC C18 separation column (75 µm × 50 cm; Thermo Scientific). The column was eluted at 0.3 µL/min with a two-step linear gradient: 4% B to 25% B over 12 min followed by 25% B to 35% B over 10 min (32 min total) and then washed with 100% B for 22 min (33 to 55 min). Effluent from the column was sprayed directly into the mass spectrometer.

Data were acquired using information directed acquisition (IDA). IDA criteria were: charge state 2–6, up to 25 fragmentation spectra per cycle, dynamic exclusion for 30 s, and maximum mass of 1800 Da. Parent ion masses were acquired in the Orbitrap (resolution 120,000), in positive mode, with a mass tolerance of 10 ppm, an automatic gain control (AGC) of 4 × 10^5^, an ion filling time of 100 ms, and a mass range of 350–1800 *m*/*z*. Fragmentation spectra were acquired in the Orbitrap (resolution 30,000), using HCD (high-energy collision-induced dissociation), with a 40% normalized collision energy, an AGC of 5 × 10^4^, a maximum ion filling time of 60 ms, and a mass range of 50 to variable *m*/*z* (top mass is set to match the parent ion *m*/*z* and charge state).

Data were interrogated for isopeptide crosslinked peptides using the Batch-Tag (Web) and Search Compare algorithms from Protein Prospector v 5.22.1 (Mass Spectrometry Facility at the University of California San Francisco, San Francisco, CA, USA). *Raw data files from the Orbitrap were converted to the *.mgf format using MSConvert (version 3.0, Proteo Wizard, Palo Alto, CA, USA). Database search was performed with Batch-Tag (Web) using the following parameters: database = SwissProt 2017.11.01; taxonomy = Homo sapiens; digest = trypsin; max missed cleavages = 3; pre-search parameters: taxonomy = Homo sapiens and accession number = P06276 (for HuBChE); constant modification = carbamidomethylation; variable modification = oxidation (M); precursor charge state = 2, 3, 4, 5; mass = monoisotopic; parent tol = 20 ppm; fragment tol = 30 ppm; instrument = ESI-Q-high-res; mass modification range = −18 to 4000; modification positions = D, E, K, protein N-term, and protein C-term; uncleaved = unchecked; crosslinking = user-defined link; link AAs = E, D, protein C-term > K, protein N-term; bridge element composition = H-2 O-1. Crosslinked peptide candidates selected by Batch-Tag were reported in Search Compare and checked by manual evaluation, with the assistance of Xcalibur/Qual Browser v 4.2.47 (Thermo Fisher Scientific).

The data also were interrogated for diethylphosphate (DEP) and *O*-ethylphosphate (MEP) adducts using Proteome Discoverer ver 2.2 (Thermo Scientific). A database search was conducted using the *.raw files from the Orbitrap. The Processing Workflow was PWF_Fusion_Basic_Sequest HT with the Fixed Value PSM Validator replaced by Percolator. The consensus workflow was CWF_Basic. The following parameters were used: database = 20190507 SwissProt—Homo sapiens; dynamic modification = oxidation Met; diethylphosphate Lys, Ser, Thr, Tyr; O-ethylphosphate Lys, Ser, Thr, Tyr; and acetyl N-terminus; static modification = carbamidomethyl Cys; enzyme = trypsin (full); missed cleavages = 2; minimum peptide length = 6; precursor mass tolerance = 10 ppm; and fragment mass tolerance = 0.02 Da.

Quantitation of peptides was based on spectral counting (i.e., the number of times a peptide appears in the mass spectral data). This method of label-free quantitation is well founded [15,16].

## Supplementary Materials

The following are available online at, Figure S1: SDS gels stained with Coomassie blue. 50 µg HuBChE per lane, Table S1: Diethylphosphate and monoethylphosphate labeled HuBChE peptides after treatment with 1.5 mM chlorpyrifos oxon ethyl.
