# Supplementary material for: Chlorpyrifos Oxon-Induced Isopeptide Bond Formation in Human Butyrylcholinesterase
Source: Molecules. 2020 Jan 25;25(3):533. doi: 10.3390/molecules25030533 (PMC7037448; doi:10.3390/molecules25030533)
Supplement: Supplementary file 1 [file molecules-25-00533-s001.pdf]

Supplementary Material

Chlorpyrifos oxon-Induced Isopeptide Bond Formation in Human Butyrylcholinesterase

Kevser Biberoglu [1], Ozden Tacal [1], Lawrence M. Schopfer [2], Oksana Lockridge [2]

1 Department of Biochemistry, School of Pharmacy, Hacettepe University, Ankara, Turkey
2 Eppley Institute, University of Nebraska Medical Center, Omaha, NE, 68198-5900

Kevser Biberoglu kevserb@hacettepe.edu.tr
Lawrence M. Schopfer lmschopf@unmc.edu
Ozden Tacal tacal@hacettepe.edu.tr
Oksana Lockridge olockrid@unmc.edu

Corresponding author
Lawrence M. Schopfer
University of Nebraska Medical Center
Omaha, NE 68198
lmschopf@unmc.edu

Figure 1S SDS gels stained with Coomassie blue. 50 μg HuBChE per lane.
Table 1S Diethylphosphate and monoethylphosphate labeled HuBChE peptides after treatment with 1.5 mM chlorpyrifos oxon ethyl

Figure 1S. Chlorpyrifos oxon ethyl treatment (panel B) converted the 85 kDa HuBChE monomer to a set of double bands in the 255 kDa region, and to a set of triplet bands in the 340-500 kDa region. Chlorpyrifos ethyl treatment (panel A) resulted in formation of a set of double bands in the 255 kDa region. Paraoxon ethyl treatment (panel D) intensified a band at 255 kDa. Diazoxon and dichlorvos treatment (panels F and H) intensified bands at 255 kDa and in the 340-500 kDa region. Treatment with methamidophos (panel C), diazinon (panel E), and monocrotophos (panel G) caused no change in band patterns.

Control HuBChE samples stored at 4˚C or at 37˚C in pH 8.5 buffer have an intense band at 85 kDa for the HuBChE monomer, a band at 170 kDa for the HuBChE dimer, a faint band at 255 kDa for the HuBChE trimer, a faint band at 340 kDa for the HuBChE tetramer, and a faint band at ~500 kDa for higher MW forms of HuBChE. Band intensities were the same for the 4˚C and 37˚C HuBChE samples.

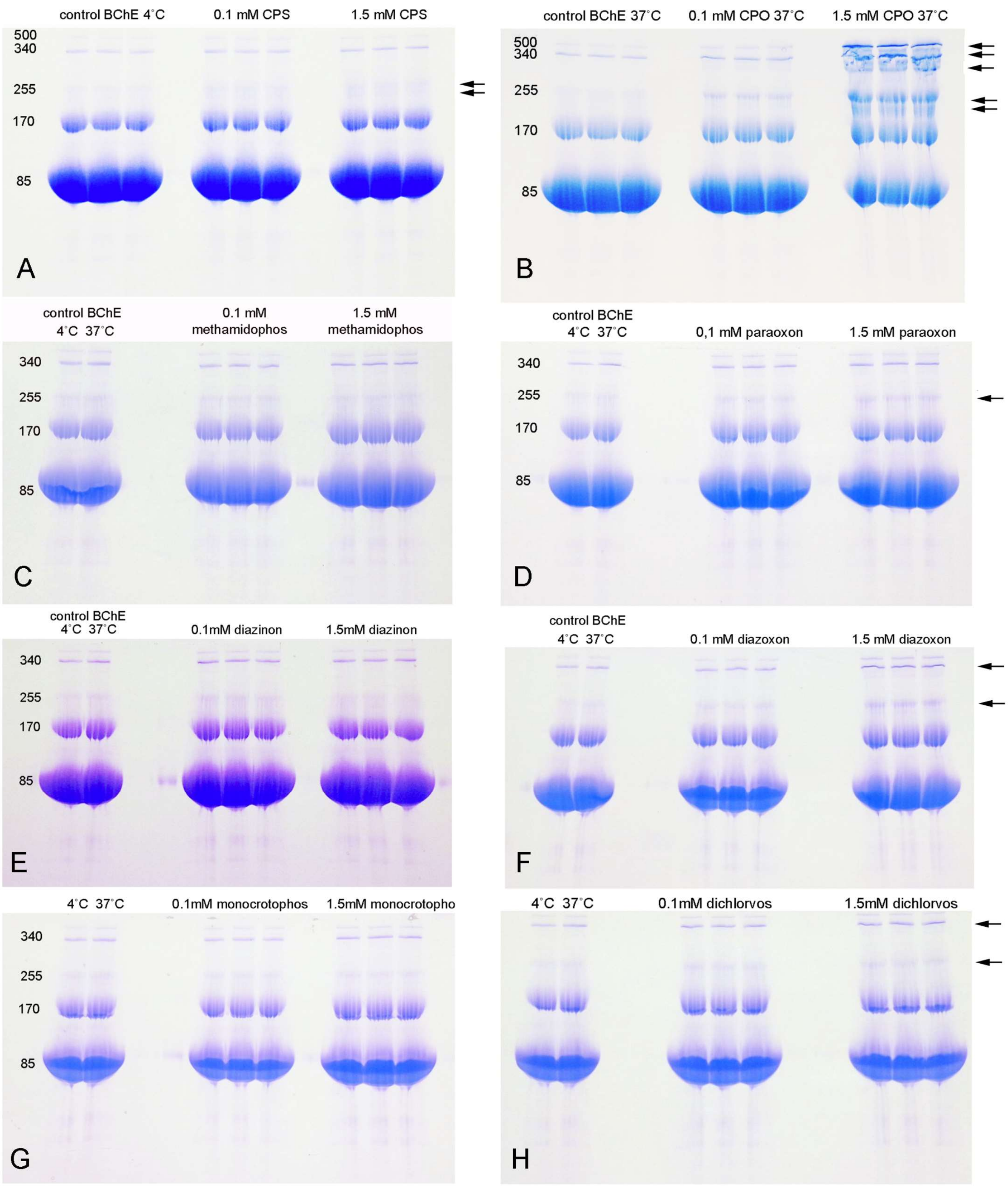

Figure 1S. SDS gels stained with Coomassie blue. 50 µg HuBChE per lane. HuBChE was incubated with 0, 0.1 mM, or 1.5 mM OP in pH 8.5 buffer at 37˚C for 7 days, or with zero OP at 4° or 37°C. Excess OP was removed by dialysis. Arrows point to bands whose intensity increased as a consequence of OP treatment. A) chlorpyrifos ethyl, B) chlorpyrifos oxon, ethyl C) methamidophos, D) paraoxon ethyl, E) diazinon, F) diazoxon, G) monocrotophos, H) dichlorvos.

Table 1S
Diethylphosphate and monoethylphosphate labeled HuBChE peptides

Peptide [A]	Labeled [B] Residue	DEP [C]	MEP [C]	Labeled [D] Count	Unlabeled [D] Count	Fraction [E] Labeled
AEEILsRSIVK	Ser 464	X		1	0	0.50
AEEILSR	-	-	-	-	1	0
AILQSGSFNAPWAVTSLyEAR	Tyr 237	X		4	13	0.20
AILQSGSFNAPWAVTsLYEAR	Ser 235	X		1	0	0.05
AILQSGSFNAPWAVTSLyEA	Tyr 237		X	3	0	0.15
DEGTAFLVyGAPGFSK	Tyr 332	X		1	13	0.07
DEGTAFLVyGAPGFSK	Tyr 332		X	1	0	0.07
ENETEIIkCamLR	Lys 262	X		1	0	1.00
ESILFHYTDWVDDQRPENyR	Tyr 385	X		3	30	0.09
ESILFHyTDWVDDQRPENYR	Tyr 373		X	1	0	0.03
FkKPQSLTK [G]	Lys 44	X		3	5	0.33
FKkPQSLTK [G]	Lys 45	X		1	0	0.10
kPQSLTK	Lys 45	X		1	0	0.10
kEFQEGLK [F]	Lys 348	X		2	1	0.50
kEFQEGLK	Lys 348		X	1	0	0.25
KFsEWGNNAFFYYFEHR	Ser 410		X	1	13	0.07
kTQILVGVNK [G]	Lys 314	X		1	2	0.11
KtQILVGVNK [G]	Thr 315	X		1	0	0.11
KTQILVGVNKDEGTAFLVYGAPGFSK	-	-		0	5	0
NQFNDyTSK [G]	Tyr 564	X		4	5	0.27
NQFNDYtSK [G]	Thr 565	X		1	0	0.27
NQFNDyTSK	Tyr 564		X	2	1	0.07
NQFNDYTSKk [G]	Lys 408	X		2	0	0.13
NQFNDYTSkK [G]	Lys 407	X		2	0	0.13
STEQkYLTLNTESTR [G]	Lys 499	X		2	2	0.33
STEQKyLTLNTESTR [G]	Tyr 500	X		2	0	0.33
SVTLFGEsAGAASVSLHLLSPGSHSLFTR [H]	Ser 198		X	14	0	1.00

Peptide [A]	Labeled [B] Residue	DEP [C]	MEP [C]	Labeled [D] Count	Unlabeled [D] Count	Fraction [E] Labeled
TLNLAkLTGCamSR	Lys 248	X		1	2	0.33
TQILVGVNKDEGTAFLVyGAPGFSK	Tyr 332	X		2	12	0.14
TQILVGVNKDEGTAFLVyGAPGFSK	Tyr 332		X	2	0	0.14
VIVVSMoxNyR	Tyr 146	X		1	13	0.07
VIVVSMoxNyR	Tyr 146		X	1	0	0.07
VIVVSMNYR	-			-	14	0
VLEMoxTGNIDEAEWEWkAGFHR	Lys 544	X		2	9	0.08
VLEMoxTGNIDEAEWEWK	-	-		-	13	0
WNNyMoxMoxDWK	Tyr 553	X		2	2	0.14
WNNyMoxMoxDWK	Tyr 553		X	1	0	0.07
WNNYMoxMDWK	-	-		-	1	0
WNNYMMoxDWK	-	-		-	1	0
WNNYMoxMoxDWkNQFNDYTSK	Lys 557	X		1	3	0.07
WNNyMoxMoxDWKNQFNDYTSK	Tyr 553		X	1	0	0.07
AGFHRWNNyMoxMoxDWK	Tyr 553	X		1	1	0.07
yLTLNTESTR [G]	Tyr 500	X		1	32	0.03
yLTLNTESTR	Tyr 500		X	1	0	0.03
YLTLNtESTR [G]	Thr 505	X		1	0	0

A Lower script indicates the labeled residue. Cysteines are carbamidomethylated and marked as Cam.

B The number following the residue is the position of the residue in the primary sequence of mature BChE (i.e. without the leader sequence).

C DEP stands for diethylphosphate; MEP stands for monoethylphosphate.

D Count refers to spectral count, which is the number to times that the peptide appeared in the mass spectral output. Spectral count has been certified as a means for determining relative amounts of peptides [Old, Meyer-Arendt, Aveline-Wolf, Pierce, Mendoza, Sevinsky, Resing & Ahn; Comparison of label-free methods for quantifying human proteins by shotgun proteomics; Mol Cell Proteomics 4, 1487 (2005)].

E Fraction labeled calculated for each labeled residue. It is the labeled spectral count divided by the sum of the labeled spectral count and the unlabeled spectral count.

F The DEP labeled lysine was Lys 349, whereas the crosslinked lysine was Lys 355.

G Protein Prospector could not decide which residue was labeled.

H This is the active site serine.
